# New insight into the catalytic -dependent and -independent roles of METTL3 in sustaining aberrant translation in chronic myeloid leukemia

**DOI:** 10.1038/s41419-021-04169-7

**Published:** 2021-09-24

**Authors:** Zaira Ianniello, Melissa Sorci, Lavinia Ceci Ginistrelli, Alessia Iaiza, Marcella Marchioni, Claudia Tito, Ernestina Capuano, Silvia Masciarelli, Tiziana Ottone, Cristina Attrotto, Manuela Rizzo, Luca Franceschini, Stefano de Pretis, Maria Teresa Voso, Mattia Pelizzola, Francesco Fazi, Alessandro Fatica

**Affiliations:** 1 Department of Biology and Biotechnology ‘Charles Darwin’, Sapienza University of Rome, Rome, Italy; 2 Department of Anatomical, Histological, Forensic & Orthopedic Sciences, Section of Histology & Medical Embryology, Sapienza University of Rome, Rome, Italy; 3 Institute of Biology, Molecular Medicine and Nanobiotechnology, CNR, Sapienza University of Rome, Rome, Italy; 4 Histology and Embryology Section, Department of Life Science and Public Health, Fondazione Policlinico Universitario A. Gemelli IRCCS, Rome, Italy; 5 Department of Biomedicine and Prevention, University of Rome Tor Vergata, Rome, Italy; 6 Fondazione Santa Lucia, Laboratorio di Neuro-Oncoematologia, Rome, Italy; 7 Fondazione Policlinico Tor Vergata, Rome, Italy; 8 Center for Genomic Science, Fondazione Istituto Italiano di Tecnologia, Milan, Italy; 9 Istituto Pasteur Italia-Fondazione Cenci Bolognetti, Rome, Italy

**Keywords:** Chronic myeloid leukaemia, Ribosome

## Abstract

Chronic myeloid leukemia (CML) is a myeloproliferative neoplasm caused by the presence of tyrosine kinase BCR-ABL1 fusion protein, which deregulate transcription and mRNA translation. Tyrosine kinase inhibitors (TKIs) are the first-choice treatment. However, resistance to TKIs remains a challenge to cure CML patients. Here, we reveal that the m^6^A methyltransferase complex METTL3/METTL14 is upregulated in CML patients and that is required for proliferation of primary CML cells and CML cell lines sensitive and resistant to the TKI imatinib. We demonstrate that depletion of METTL3 strongly impairs global translation efficiency. In particular, our data show that METTL3 is crucial for the expression of genes involved in ribosome biogenesis and translation. Specifically, we found that METTL3 directly regulates the level of PES1 protein identified as an oncogene in several tumors. We propose a model in which nuclear METTL3/METTL14 methyltransferase complex modified nascent transcripts whose translation is enhanced by cytoplasmic localization of METTL3, independently from its catalytic activity. In conclusion, our results point to METTL3 as a novel relevant oncogene in CML and as a promising therapeutic target for TKI resistant CML.

## Introduction

Chronic myeloid leukemia (CML) is associated in about 95% of patients with a translocation between chromosome 9 and 22 that results in the production of the oncogenic BCR-ABL1 fusion gene [1]. Abl1 is a protein tyrosine kinase that is constitutively activated in the BCR-ABL1 fusion resulting in the alteration of multiple signaling pathways that regulate gene expression [2]. The use of ABL1 tyrosine kinase inhibitors (TKIs) made CML clinically manageable and curable. However, treatment with TKIs is not always curative. About 20% of CML patients will develop resistance, and in patients with myeloid blast crisis TKIs will produce only short-term responses [3–5].

The N^6^-methyladenosine (m^6^A) modification in mRNA is an important driver of malignant transformation in the hematopoietic compartment [6–8]. Although progress has been made in the understanding of signal transduction in the BCR-ABL1-mediated transformation, the role of m^6^A in CML remains unknown. The major complex responsible for m^6^A modification within mRNA is the METTL3/METTL14 complex [9]. METTL3 is the only catalytic subunit while METTL14 is required for RNA binding [9]. Moreover, in lung cancer METTL3 localized to the cytoplasm, where it can act as m^6^A reader and promotes translation of m^6^A modified mRNAs [10, 11].

Here, we show that the m^6^A methyltransferase complex METTL3/METTL14 is upregulated in primary CML samples, and that its downregulation strongly affects proliferation of both primary CML and imatinib -sensitive and -resistant CML cell lines. We demonstrate that METTL3 silencing in CML cells strongly affects ribosomal particle levels and global translation efficiency. Notably, aberrant translation is considered one of the mechanisms that mediate BCR/ABL transformation and sustain the leukemic phenotype of CML cells [12, 13]. Here, we demonstrate that METTL3 regulates ribosome levels and translation indirectly, by affecting MYC levels, and directly, by methylating specific mRNAs and stimulating their translation. In the latter case, we show that the impact of METTL3 on mRNA translation depends on its localization in the cytoplasm and is independent from its catalytic activity. Thus, we propose a model in which nuclear METTL3/METTL14 methyltransferase complex modified nascent transcripts whose translation is enhanced by cytoplasmic METTL3. In particular, we found that METTL3 directly regulates the level of *Pescadillo (*PES1) protein, which was shown to be a crucial regulator of ribosome biogenesis and cell proliferation, and a key factor in cell cycle progression in different tumors [14–18]. These results indicate that catalytic -dependent and -independent roles of METTL3 support aberrant translational of CML cells. Furthermore, we revealed the presence of a new oncogenic axis involving METTL3 and PES1. Our data suggest that the inhibition of the METTL3/METTL14 complex may represent an effective therapy for killing CML cells that evade tyrosine kinase inhibition.

## Materials and methods

### Cell cultures

K562 and K562 imatinib-resistant cell lines (the American Type Culture Collection), KCL22, LAMA84, NB4, HL-60, HEL and U937 cell lines were cultured in RPMI 1640 medium with 1x Penicillin/Streptomycin solution, 1x L-glutamine and 10% Fetal Bovine Serum (FBS) at 37 °C under an atmosphere containing 5% CO_2_. Cell lines were routinely tested for mycoplasma contamination (LookOut Mycoplasma PCR Detection Kit, Merck KGaA, Darmstadt, Germany). Cycloheximide and Imatinib mesylate were purchased from Thermo Fisher Scientific, Waltham, MA USA. CML primary samples were collected at diagnosis from peripheral blood at the Department of Biomedicine and Prevention, University of Rome Tor Vergata, after obtaining written informed consent to the study from all patients and approval of the study by the IRB of Policlinico Tor Vergata, Rome (IT). Mononuclear cells were isolated using Ficoll-Paque density gradient centrifugation. CML cells were cultured in StemSpan™ Leukemic Cell Culture Kit (STEMCELL Technologies, Tukwila, WA, USA).

### Cell proliferation analysis

Data for growth curves and cell viability were obtained with the Countess II FL Automated Cell Counter (Thermo Fisher Scientific, Waltham, MA, USA), excluding the trypan blue positive cells. Cell cycle was analyzed by flow cytometry (CyAN ADP DAKO, Beckman Coulter, Brea, CA, USA) as previously described [19].

### Lentiviral production

Lentiviruses were prepared from mission pLKO.1 Lentiviral shRNA clones (Merck KGaA, Darmstadt, Germany) TRCN0000289812 (shMETTL3), TRCN0000289814 (shMETTL3_2), TRCN0000015937 (shMETTL14) and SHC202 TRC2 (Non-Target shRNA Control) as described [20]. Transduction and selection were performed as previously described [21].

### Immunoblot analysis

Immunoblot analysis nuclear/cytoplasmic fractionation were performed as previously described [21].

### Polysome profiling

Cytoplasm fractionations on sucrose gradients were performed as described [21]. 35 μl of each fraction were pooled together in four fractions (Heavy Polysomes, Light Polysomes, 40 S/60 S, Free RNA). Global protein synthesis was measured by the SUnSET method [22].

### RNA sequencing

Total RNA was extracted using the miRNeasy Mini Kit (Qiagen, Hilden, Germany). Total RNA from triplicates was sent to I.G.A. Applied Genomics Institute (Udine, IT) for library preparation using the TruSeq stranded mRNA kit (Illumina San Diego, CA, USA) and subjected to sequencing (125 bp paired-end). The RNA-seq data were analyzed with the Artificial Intelligence RNA-seq Software as a Service (SaaS) platform (https://transcriptomics.cloud). Quality was assessed using FastQC. Bad quality reads were removed using BBDuk (http://sourceforge.net/projects/bbmap/) by setting a minimum length of 35 bp and a minimum Phred-quality score of 25. Afterwards, high quality reads were mapped against the reference genome with STAR [23] using the end-to-end alignment mode and gene expression quantification was performed with featureCounts [24]. The statistical analysis started by filtering lowly expressed genes using HTSFilter [25]. DESeq2 software was used for differentially gene expression [26]. Finally, GOEA was performed with in-house scripts based on hypergeometric tests [27].

### m^6^A-Seq

Total RNA was isolated from shSCR and shMETTL3 K562 cells 9 days after transduction. PolyA+ RNA was purified using the GenElute™ mRNA Miniprep Kit (Merck KGaA, Darmstadt, Germany). 5 μg of polyA+ RNA were used for each immunoprecipitation. The m^6^A-Seq was performed using the Magna MeRIP m^6^A kit (Merck KGaA, Darmstadt, Germany). Immunoprecipitated RNA fragments were sent to the NGS facility of the Centre for Integrative Biology (CIBIO), University of Trento (IT), for library preparation using the NEB Ultra II RNA directional kit (New England BioLabs, Ipswich, MA USA) and subjected to sequencing. We used HTS-flow [28] for the filtering, quality checks and alignment of the reads. ExomePeak [29] was used to call peaks (cutoff FDR 0.05) in the two conditions (shSCR and shMETTL3). Peaks were further selected by discarding peaks with low number of reads (bottom 20th). Genes associated with the m^6^A peaks in each condition were retrieved using the compEpiTools package. Enrichment analysis (p-value cutoff 1e-10) based on the GO biological process ontology was used to identify the most represented gene functions. GO processes associated with more than 3 K genes (exceedingly general terms) and less than 10 genes (exceedingly specific terms) were discarded. Redundancy between the selected terms was minimized discarding parents of children terms with more than 50% overlap in the gene list. m6A peaks distribution and enriched motifs analysis were performed with RNAmod (https://bioinformatics.sc.cn/RNAmod/index.php). Peaks validations were performed as previously described [21].

### Cross-linking immunoprecipitation

Cross-linking immunoprecipitation in K562 cells stably expressing dox inducible FLAG-METTL3 or FLAG-METTL3 APPA were performed as previously described [21].

## Results

### The METTL3/METTL14 complex is upregulated in CML

**Fig. 1:**
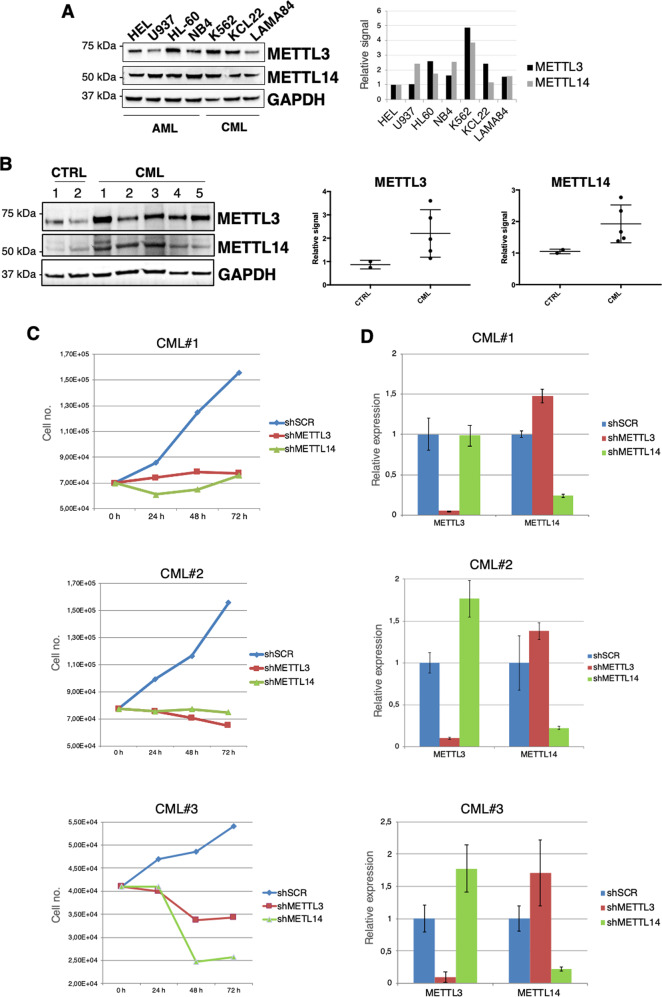
METTL3 and METT14 proteins are upregulated in CML and required for primary CML cells viability. **A** Left panel, Western blot analysis of METTL3 and METTL14 AML (HEL, HL-60, NB4, U937) and CML (K562, KCL22, LAMA84) cell lines. Right panels, ﻿the histogram represents densitometric analysis of METTL3/GAPGH and METTL14/GAPDH ratio from the western blot analysis showed on the side. **B** Left panel, Western blot analysis of METTL3 and METTL14 levels in primary CML samples and peripheral blood mononuclear cells from healthy donors. Right panels, ﻿the dot blots represent densitometric analysis of METTL3/GAPGH and METTL14/GAPDH ratio from the western blot analysis showed on the side. Mean and standard deviation are indicated. **C** Growth curve of primary CML infected cells after puromycin selection. **D** Expression levels of METTL3 and METTL14 transcripts in primary CML cells infected with shSCR, shMETTL3 and shMETTL14 lentivirus performed at 48 h after puromycin selection. Values are normalized against ACTB and expressed as relative quantities with respect to shSCR cells set to a value of 1. RNA quantity in the bars is represented as the mean of the fold change with standard deviation.

METTL3 and METTL14 are upregulated in acute myeloid leukemia (AML) where they have an established oncogenic function and play a relevant role in AML survival [30–32]. Notably, expression levels of the m^6^A methyltransferases METTL3 and METTL14 in CML cell lines are comparable if not higher, as in the case of K562 cells, than that of AML cell lines (Fig. 1A). However, the function of the METTL3/METTL14 complex in CML is still unknown. Thus, we examined METTL3 and METTL14 protein levels in a panel of primary BCR-ABL1^+^ CML and healthy control samples. We observed significant upregulation of both proteins in CML patients (Fig. 1B). These data indicate that the METTL3/METTL14 complex might have an oncogenic role in CML.

To study the contribution of the m^6^A methylation complex in CML, we performed METTL3 and METTL14 knock-down in three different primary CML samples. Non-targeting scramble shRNA (shSCR) was utilized as control. Strikingly, silencing of the m^6^A methylation complex strongly impaired CML proliferation (Fig. 1C and D).

### METTL3 and METTL14 are required for proliferation of CML cells

**Fig. 2:**
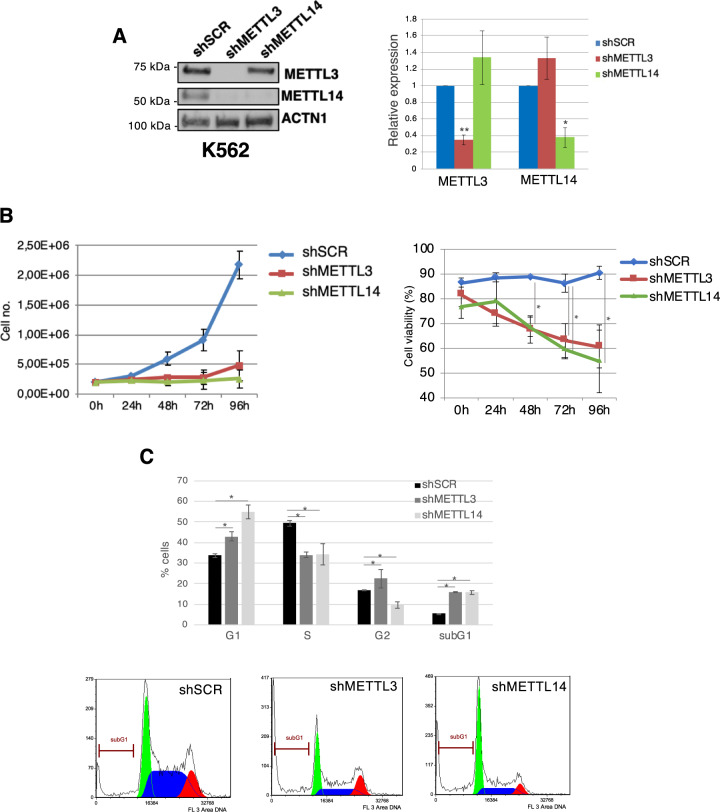
METTL3 and METTL14 knockdown affects K562 cells proliferation. **A** Left panel: Western blot analysis of METTL3 and METTL14 levels in shMETTL3, shMETTL14 and shSCR K562 transduced cells. Protein levels were analyzed 48 h after puromycin selection. Right panel: expression levels of METTL3 and METTL14 mRNA in shSCR, shMETTL3 and shMETTL14 cells. Values are normalized against ACTB and expressed as relative quantities with respect to shSCR cells set to a value of 1; *n* = 3. Relative RNA quantity in the bars is represented as the mean of the fold change with standard deviation. **B** Growth curve (left panel) and percentage of cell viability (right panel) of K562 infected cells after puromycin selection, *n* = 3. **C** Left panel, the histogram represents the cell cycle distribution of cells analyzed 48 h after puromycin selection, *n* = 3. Right panel, representative cell cycle analysis. The ratio of each sample versus its experimental control was tested by two-tailed Student’s *t*-test. *p < 0.05, ** *p* < 0.01.

**Fig. 3:**
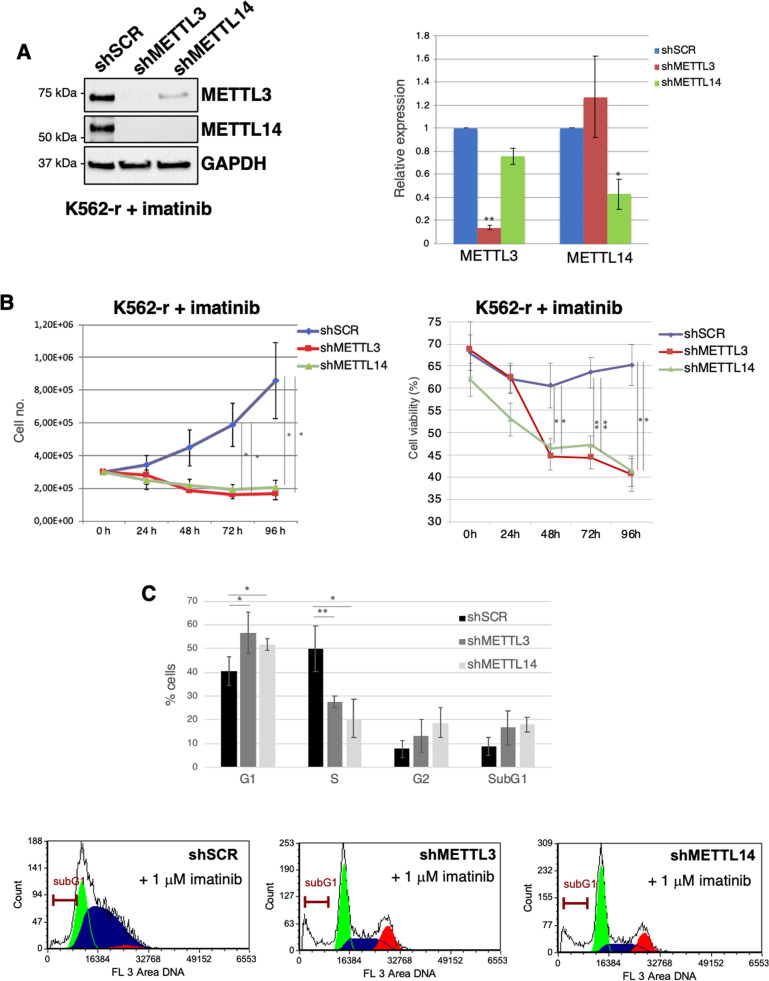
METTL3 and METTL14 knockdown affects imatinib-resistant K562 cells proliferation. **A** Western blot analysis in shMETTL3, shMETTL14 and shSCR K562-r transduced cells. Protein levels were analyzed 48 h after puromycin selection. **B** Growth curve (left panel) and percentage of cell viability (right panel) of shSCR, shMETTL3 and shMETTL14 K562-r infected cells after puromycin selection, *n* = 5. **C** Upper panel, the histogram represents the cell cycle distribution of cells analyzed 48 h after puromycin selection, *n* = 3. Lower panel, representative cell cycle analysis. The ratio of each sample versus its experimental control was tested by two-tailed Student’s *t-*test. * *p* < 0.05, ** *p* < 0.01.

To assess in more detail the oncogenic role of m^6^A modification in CML, we performed METTL3 and METTL14 silencing in the BCR-ABL1^+^ CML cell line K562 (Fig. 2). Western blot analysis showed significant downregulation of METTL3 and METTL14 proteins (Fig. 2A), which results in impaired cell growth and cell viability (Fig. 2B). Cell cycle analysis showed an increase in G1 and a decrease of S phase (Fig. 2C). Moreover, we also observed a slight increase in sub-G1 apoptotic population (Fig. 2C). Cell division was also monitored by eFluor labeling and, also in this case, silencing of METTL3 and METTL14 strongly affects the distribution of the fluorescence intensity in the daughter cells indicating a strong impairment in proliferation (Supplemental Figure S1). We next investigated the effect of METTL3 and METTL14 knockdown on the TKI imatinib mesylate (imatinib) -resistant K562 cell line (K562r) (Fig. 3). The proliferation of K562r control cells (shSCR) were not inhibited despite a high dose of imatinib (1 μM) (see IC50 in Supplemental Fig. S2). Notably, in METTL3 and METTL14 interfered cells we observed a robust reduction of proliferation potential and cell viability with a strong reduction of S phase and increase of G1 phase of the cell cycle (Fig. 3B and C). Results in imatinib sensitive and resistant K562 cells were also confirmed with a second shRNA against METTL3 (Supplemental Figs. S3 and S4). These data showed that the m^6^A methylation complex is required for supporting the survival of imatinib -sensitive and -resistant K562 cells. We also performed METTL3 knockdown in the BCR-ABL1^+^ CML cell lines KCL22 and LAMA84. Also in this case, the depletion of METTL3 strongly impaired cell growth and cell viability (Supplementary Figs. S5 and S6).

### RNA-seq and m^6^A-seq in K562 cells

**Fig. 4:**
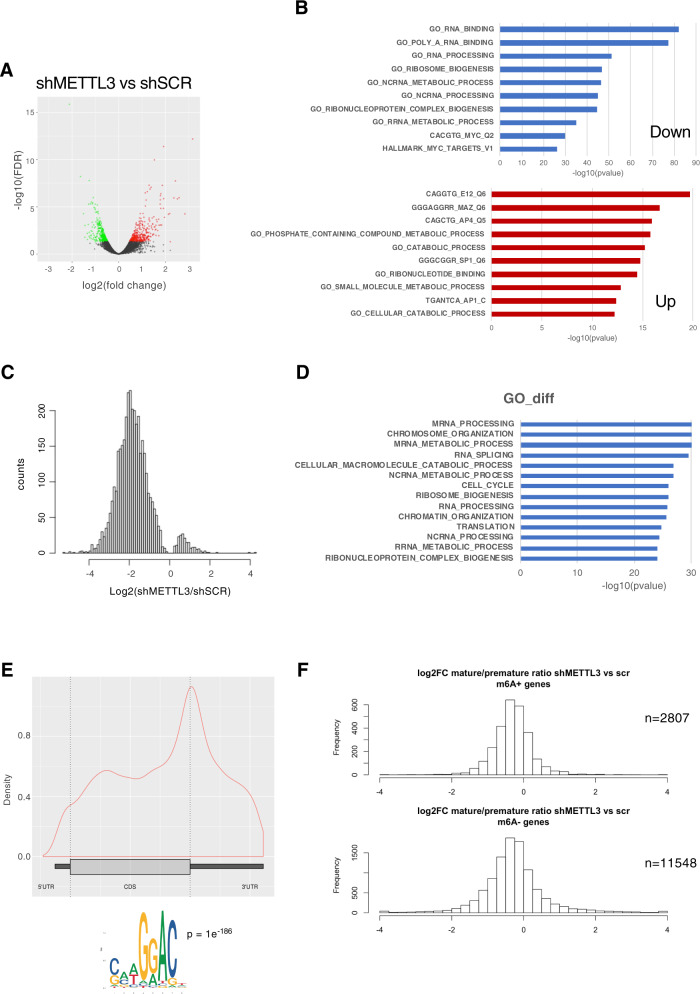
RNA expression and m^6^ A distribution in K562 cells upon METTL3 knockdown. **A** RNA-seq was performed on shSCR and shMETTL3 K652 cells from triplicates. The Volcano plot shows the relationship between the fold-change and the significance of the differential expression test for each gene in the genome in shMETTL3 vs shSCR. Black dots represent the genes that are not significantly differentially expressed, while red and green dots are the genes that are significantly up- and down-regulated, respectively. **B** The top 10 GO terms associated with biological processes of differentially expressed genes are shown. **C** Histogram showing the changes in m^6^A enrichment between shMETTL3 and shSCR control cells. **D** Enrichment analysis (*p*-value cutoff 1e-10) based on the GO biological process ontology was used to identify the most represented gene functions in m^6^A marked genes. **E** Upper panel, distribution of m^6^A peaks within the 5’-UTR, CDS, and 3’-UTR regions of mRNAs from shSCR m6A-seq. Lower panel shows the most enriched sequence motif of m^6^A peaks. **F** The histograms represent the ratio between mature RNA expression and premature RNA expression in shMETTL3 vs shSCR cells in differentially m^6^A methylated genes (m6A+ and m6A−).

To identify the regulatory role of the m^6^A methylation complex in K562 cells, we performed RNA sequencing on cells interfered for the catalytic subunit METTL3 (shMETTL3) (Fig. 4). We found 173 genes significantly downregulated and 133 genes significantly upregulated in shMETTL3 vs shSCR (Fig. 4A, Supplemental Table S1). Biological process ontology of significantly differentially expressed genes identified several pathways including RNA binding, RNA processing and ribosome biogenesis (Fig. 4B). Thus, these data indicate that genes involved in RNA metabolism are significantly affected in CML cells depleted of METTL3.

To test whether the altered gene expression could be a consequence of m^6^A demethylation, we compared the m^6^A distribution in shSCR and shMETTL3 cells by m^6^A-sequencing (m^6^A-Seq). We identified 3710 differential peaks in SCR vs shMETTL3 cells (Supplementary Tables S2 and S3), 3517 of which have a negative variation upon METTL3 silencing (Fig. 4C and Supplementary Fig. S7). As previously reported, m^6^A peaks within mRNAs were enriched near stop codons and 3’-UTRs and occurred within the consensus RRACH motif (R = A or G; H = except G; Fig. 4E). GO biological process ontology was used to identify the most represented gene functions. A number of processes related to RNA processing, ribosome biogenesis and translation were identified with high significance in differentially m^6^A methylated genes (Fig. 4C). We then asked whether differential m^6^A methylation was associated with changes in post-transcriptional regulation in the same conditions. In order to estimate this, we used INSPEcT to calculate the ratio between mature RNA expression (~ exons) and premature RNA expression (~ introns) [33, 34]. At steady state, this ratio corresponds to the ratio between the rate of premature RNA processing and the rate of mature RNA degradation (Furlan et al., [34]). Thus, a change in this ratio between two conditions indicates that one or both these processes are affected. We determined the ratio of shMETTL3 considering the 2807 genes whose transcripts were differentially methylated in shMETTL3, compared to shSCR. While for shMETTL3 the ratio decreased (one-tailed Wilcoxon test *P* < 1e-185; Fig. 4F), the same trend occurred for 11548 genes whose transcripts are not differentially methylated (one-tailed Wilcoxon test *P* < 2e-95). Thus, at the time of the analysis, while an important fraction of the differentially methylated genes is likely to be post-transcriptionally regulated, this is unlikely to be directly driven by m^6^A depletion.

### METTL3 is required for efficient mRNA translation in CML cells

**Fig. 5:**
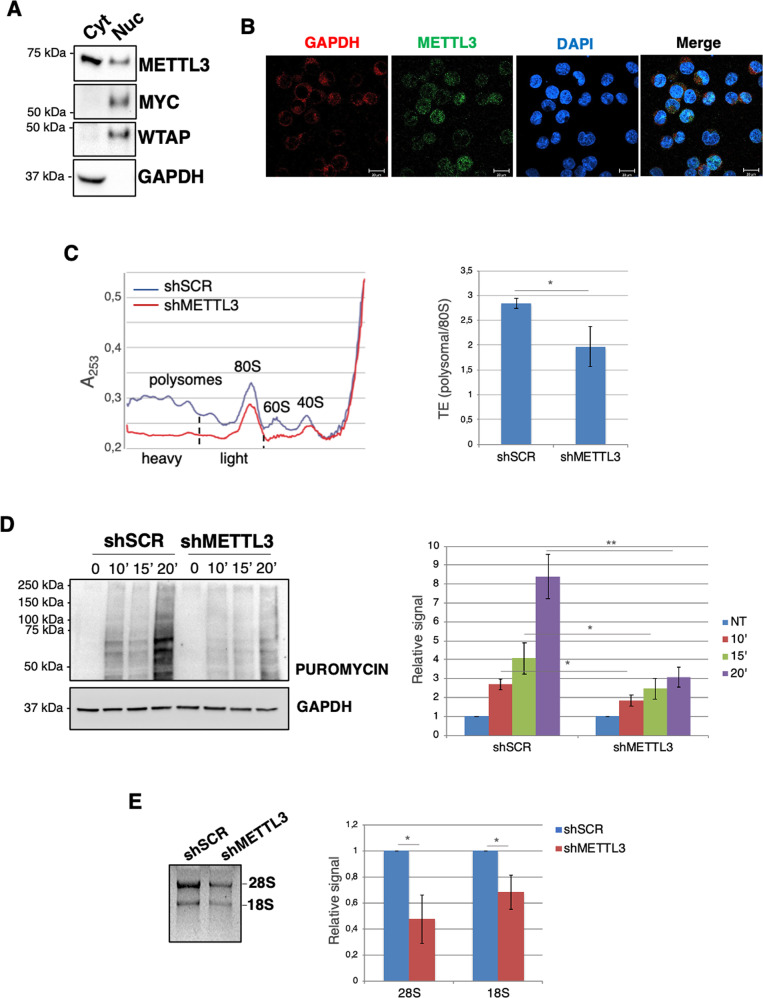
METTL3 localised in the cytoplasm of CML cells and is required for efficient translation. **A** Representative Western blot analysis from cytoplasmic (cyt) and nuclear (nuc) fractions from K562 cells. **B** Immunofluorescent analysis of endogenous METTL3 in K562 cells. GAPDH and DAPI indicated the cytosolic and nuclear compartment, respectively. Scale bar, 20 mm. **C** Cytoplasmic extracts from shSCR and shMETTL3 cells were loaded on 15–50% sucrose gradients and fractions measured by absorbance at 253 nm. Left panel: representative sucrose gradient profile out of three independent biological replicates from control and METTL3 knockdown cells. Right panel: the histogram represents the calculation of the global translation efficiency (TE) in shSCR and shMETTL3 cells. **D** SUrface SEnsing of Translation (SUnSET) was utilized to measure global protein synthesis rates in shSCR and shMETTL3 cells. Left panel, representative image of Western Blot analysis for puromycin detection. GAPDH was used as internal control. Right panel, the histogram represents the mean with standard deviation of the fold change of relative signal with respect to non-treated cells (NT) set to a value of 1; *n* = 3. **E** Left panel, representative agarose gel for the analysis of the 28 S and 18 S rRNAs expression. Total RNA was extracted from the same number of shSCR and shMETTL3 cells. Right panel, the histogram represents the mean with standard deviation of the fold change of relative signal with respect to shSCR cells set to a value of 1; *n* = 3. The ratio of each sample versus its experimental control was tested by two-tailed Student’s *t*-test. * *p* < 0.05.

METTL3 can also be localized in the cytoplasm and acts as an m^6^A reader to stimulate mRNA translation [17, 18]. We detected a consistent localization of METTL3 in the cytoplasm of CML cells (Fig. 5A and B; Supplemental Fig. S8). Interestingly, the depletion of METTL3 has a strong effect on METTL4 protein levels, without affecting METTL14 mRNA levels, indicating that the association with METTL3 in the nuclear m^6^A methylation complex is required for METTL14 stability (Figs. 2 and 3). On the other hand, METTL3 protein is still present upon METTL14 knockdown. Thus, this might be due to the METTL14-independent cytoplasmic localization of METTL3 protein [17].

Since aberrant translation contributes to the phenotype of BCR/ABL-transformed cells, we analyzed the effect of METTL3 knockdown on global translation by polysome profiling in K562 cells (Fig. 5C). We observed a strong decrease of 40 S, 60 S, 80 S and polysomes. Moreover, we assessed the global translation efficiency (TE) of the cells by calculating the ratio between the absorbance of polysomes and the total absorbance of non-translating 80 S ribosomes, as described [35]. METTL3 knockdown resulted in a significantly reduced global TE (Fig. 5C). To further analyze the role of METTL3 on protein synthesis, we examined the synthesis of nascent polypeptides using puromycin incorporation assay in shMETTL3 K562 cells, which provides a measure of the global translation efficiency in cells [22]. We observed a strong decrease in protein synthesis upon METTL3 knockdown (Fig. 5D). Furthermore, in agreement with the decrease of mature 40 S and 60 S ribosomal particles, METTL3 silencing strongly affected levels of mature 28 S and 18 S ribosomal RNAs (Fig. 5E). Results were also confirmed with a second shRNA against METTL3 (Supplemental Fig. S9). Altogether, these data indicate that METTL3 plays a crucial role in sustaining ribosome levels and translational potential of CML cells.

### METTL3 regulates translation by direct and indirect effects

**Fig. 6:**
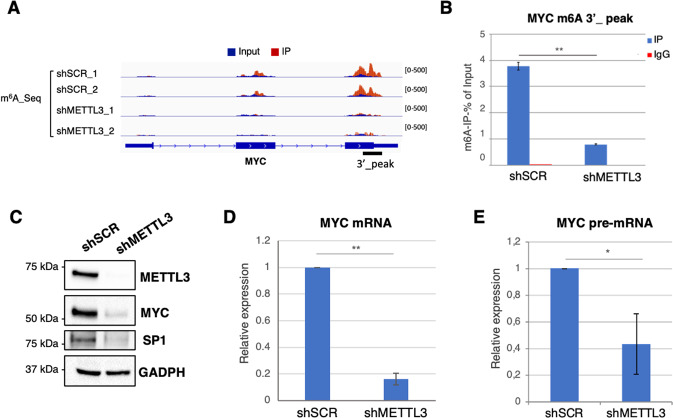
METTL3 regulates MYC levels in CML. **A** The m^6^A abundances on MYC mRNA in shMETTL3 and control shSCR cells as detected by m^6^A-seq. **B** m^6^A-IP-qPCR validation of MYC m^6^A peaks in shSCR and shMETTL3 cells. Values are expressed as percentage of input. IgG was used as control; *n* = 3. **C** Western blot analysis in shMETTL3 and shSCR K562 transduced cells. Protein levels were analyzed 48 h after puromycin selection. **D**, **E** Expression levels of MYC mRNA (**D**) and MYC pre-mRNA (**E**) in shSCR and shMETTL3 cells. Values are normalized against ACTB and expressed as relative quantities with respect to shSCR cells set to a value of 1; *n* = 3. In all panels, relative RNA quantity in the bars is represented as the mean of the fold change with standard deviation. The ratio of each sample versus its experimental control was tested by two tailed Student’s *t*-test. *p < 0.05, **p < 0.01.

We also observed downregulation of MYC-regulated genes in the RNA-seq analysis (Fig. 4B). MYC is a well-known target of the METTL3/METTL14 methylation complex and is a transcriptional activator of genes involved in ribosome biogenesis and RNA processing [36]. We confirmed m^6^A methylation of the MYC transcript by m^6^A-Seq and m^6^A immunoprecipitation (m^6^A-IP) and observed reduced m^6^A methylation in METTL3 knockdown cells (Fig. 6A and B). The METTL3/METTL14 complex regulates MYC expression at different levels including mRNA stability, translation [31, 32] and, indirectly, transcription through modulation of the SP1 transcription factor [30]. More recently, METTL3 was also shown to directly regulate MYC mRNA stability and translation in normal hematopoietic stem cells [30, 33]. Thus, we analyzed MYC levels upon METTL3 silencing in K562 cells. In shMETTL3 cells, we observed strong downregulation of both MYC protein, mRNA levels and pre-mRNA levels (Fig. 6C–E, Supplemental Fig. S3). We also observed downregulation of the MYC transcriptional regulator SP1. Altogether, these data indicate that in CML METTL3 controls MYC expression at multiple levels; thus, indicating that the downregulation of genes involved in RNA metabolism observed in the RNA-seq is due, at least in part, to the loss of MYC transcriptional activity. Indeed, most of these genes presented MYC binding in the promoter region in K562 cells (Supplementary Fig. S10).

**Fig. 7:**
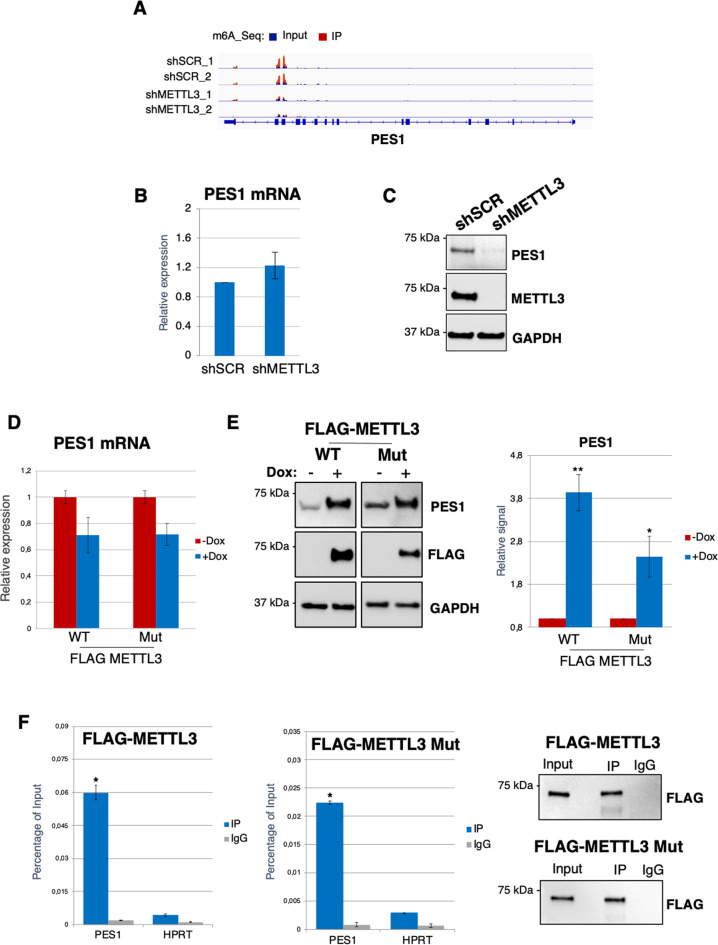
METTL3 regulates PES1 translation. **A** The m^6^A abundances on PES1 transcripts in shMETTL3 and control shSCR cells as detected by m^6^A-seq. **B** Expression levels of PES1 mRNAs in shSCR and shMETTL3 cells. Values are normalized against ACTB and expressed as relative quantities with respect to shSCR cells set to a value of 1; *n* = 3. **C** Western blot analysis in shMETTL3 and shSCR K562 transduced cells. Protein levels were analyzed 48 h after puromycin selection. **D** qRT-PCR analysis of PES1 mRNA expression in K562 cells overexpressing METTL3 and METTL3 catalytic inactive mutant (METTL3 Mut). **E** Western blot analysis of PES1 and METTL3 in the same cell of panel **D**. Densitometric analysis of PES1/GAPDH ratio from biological replicates (*n* = 3) is shown below with s.e.m. **F** Left panel, qRT-PCR of CLIP experiments performed with FLAG-METTL3 and FLAG-METTL3 Mut from cytoplasmic extract of K562 cells. Right panel, representative Western blot analysis of FLAG-tagged proteins IP *vs* Input (1%) and control IgG. In all panels, relative RNA quantity in the bars is represented as the mean of the fold change with standard deviation. Ratio of each sample versus its experimental control was tested by two tailed Student’s *t*-test. *p < 0.05, **p < 0.01.

Upon METTL3 knockdown we also observed a concomitant loss of m^6^A methylation in genes involved in ribosome biogenesis and translation (Fig. 4D). This suggests that the observed phenotype might not be only due to the loss of MYC activity. The downregulation in the ribosome biogenesis process and the concomitant arrest of the cell cycle in G1 phase, prompted us to investigate PES1 protein [15]. PES1 was shown to be involved in the maturation of the 60 S ribosomal subunit [37] and in the progression of the cell cycle [14]. In particular, we identified PES1 as relevant m^6^A methylated genes by METTL3 (Fig. 7A). Notably, despite the strong decrease of m^6^A levels in PES1 mRNA upon METTL3 knockdown, PES1 mRNA levels are not affected by the loss of METTL3. Nevertheless, we observed a strong downregulation of PES1 protein levels (Fig. 7B and C, Supplemental Fig. S3). Thus, our data indicate that PES1 depletion is not due to the loss of MYC transcriptional activity but to the loss of m^6^A methylation. PES1 regulation by METTL3 was also confirmed in K562r, KCL22 and LAMA84 CML cells (Supplementary Figs. S5, S6 and S11).

In view of these results, we hypothesized that METTL3 might directly regulate PES1 mRNA translation. By using K562 cells stably expressing doxycycline (dox) inducible METTL3 or METTL3 catalytic inactive mutant (aa395–398, DPPW → APPA, METTL3 Mut), we showed that both METTL3 isoforms positively regulate PES1 protein levels without increasing its mRNA levels (Fig. 7D and E). The binding of METTL3 and METTL3 Mut to PES1 mRNA in the cytoplasm of K562 cells was confirmed by CLIP experiments (Fig. 7F). Thus, cytoplasmic METTL3 positively controls mRNA translation independently from its catalytic activity in CML cells.

These data indicate that METTL3 regulates ribosome levels and translation indirectly, by affecting MYC levels, and directly, by methylating specific mRNAs, such as PES1, and stimulating its translation. In the latter case, both catalytic -dependent and -independent roles are required.

## Discussion

CML patients are characterized by aberrant BCR-ABL1 tyrosine kinase activity [1]. TKIs, such as imatinib, block the constitutively activated Bcr-abl1 protein and can control the aberrant proliferation of leukemia cells. After some months on TKI-therapy, most patients with CML achieve complete remission. However, inhibition of BCR-ABL1 fails to eradicate the disease in at least one-third of patients. In this case, the disease can progress to the acute phase, also referred to as blast crisis, characterized by uncontrolled proliferation of leukemic cells, similar to AML. Thus, it is not surprising that CML studies are focusing on developing novel treatments to use in combination, or in alternative to TKIs.

Oncogenic BCR-ABL1 deregulates signaling pathways controlling transcription and mRNA translation. The latter results from the positive effect of BCR-ABL1 on the translation machinery and the expression of regulators of mRNA translation [12].

**Fig. 8:**
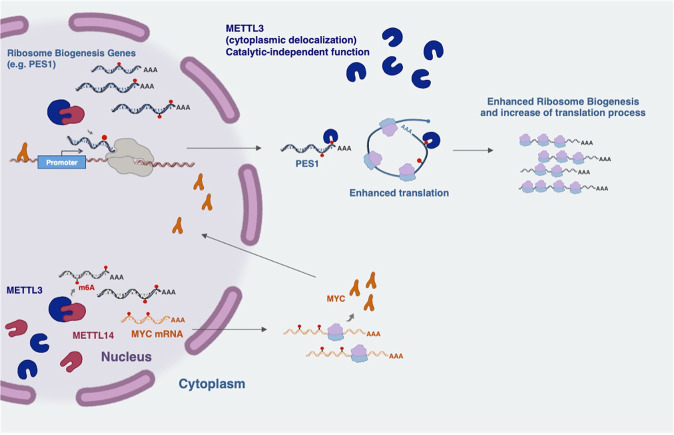
Schematic model for the role of METTL3 and METTL14 in CML. METTL3 acts in the nucleus in combination with METTL14 to modify nascent transcripts whose translation can be enhanced by the binding of METTL3 in the cytoplasm.

Here, we demonstrate that the METTL3/METTL14 complex plays a crucial role in the proliferation potential of both primary CML cells and CML cell lines. We show that downregulation of METTL3 and METTL14 overcomes the resistance of CML cells to the TKI imatinib mesylate (imatinib). Moreover, our data indicate that METTL3 sustains ribosome levels and translation by acting in the nucleus in combination with METTL14 to modify nascent transcripts whose translation is enhanced by cytoplasmic METTL3 localization (Fig. 8). Moreover, we identify PES1 as a relevant METTL3 target. Notably, the mechanism by which m^6^A modification regulates mRNA translation is still not clear. Initially, the YTHDF1 and YTHDF3 m^6^A readers were demonstrated to promote translation of m^6^A-containing transcripts via recruiting the translation initiation complex. However, recent studies showed that YTHDF proteins behaved redundantly to regulate mRNA degradation [38–40] and their involvement in mRNA translation is currently under debate. Interestingly, METTL3 was initially identified as a cytoplasmic m^6^A reader in lung cancer and it was shown to promote translation of a subset of modified mRNAs by promoting mRNA circularization [11]. In acute myeloid leukemia (AML), METTL3 and METTL14 have been already described as regulators of mRNA translation. However, for METTL3 this effect was shown to depend on relief of ribosome stalling at GAN codons containing m^6^A [30]; while for METTL14, it was shown that the regulation of translation was not mediated by YTH readers [32]. Here, we propose that the translation-promoting effect by METTL3 on m^6^A-modified mRNAs is a general mechanism that can be applied to all cells where METTL3 localized to the cytoplasm.

Moreover, our data reveal that the use of inhibitors of the METTL3/METTL14 complex may represent an effective therapy for killing CML cells that evade tyrosine kinase inhibition. Notably, conditional knockout of METTL3 in the adult mouse hematopoietic system produced an expansion of the HSCs in bone marrow without significantly affecting mature myeloid cell function [41, 42]. Recently, a chemical inhibitor of METTL3 has been developed that presents potent antileukemic effect and it has no effect on normal hematopoietic stem cells [43].

## Supplementary information

Supplemental materials and methods

Supplemental Figure S1

Supplemental Figure 2

Supplemental Figure 3

Supplemental Figure 4

Supplemental Figure 5

Supplemental Figure 6

Supplemental Figure 7

Supplemental Figure 8

Supplemental Figure 9

Supplemental Figure 10

Supplemental Figure 11

Supplemental Table 1A

Supplemental Table 1B

Supplemental Table 2

Supplemental Table 3

## Data Availability

Data and materials are available through the accession number PRJNA533293 in NCBI and the corresponding authors on request.
