# Supplementary material for: New insight into the catalytic -dependent and -independent roles of METTL3 in sustaining aberrant translation in chronic myeloid leukemia
Source: Cell Death Dis. 2021 Sep 24;12(10):870. doi: 10.1038/s41419-021-04169-7 (PMC8463696; doi:10.1038/s41419-021-04169-7)
Supplement: Supplementary file 1 — Supplemental materials and methods [file 41419_2021_4169_MOESM1_ESM.docx]

**Supplemental Meterials and Methods**

**Oligonucleotides used in this study**

ACTB_hs_FW: CGTACCACTGGCATCGTGAT

ACTB_hs_REV: GTAGTCAGTCAGGTCCCGGC

METTL3_FW: AAGCAGCTGGACTCTCTGCG

METTL3_REV: GCACTGGGCTGTCACTACGG

METTL14_FW: GCAGTTGGGAGCTGAAAGTG

METTL14_REV: GGAAGCCCTGCAAGTTTCTC

pre-MYC_Int1_FW: TAACTCAAGACTGCCTCCCG

pre-MYC_Ex2_REV: CCTCCTCGTCGCAGTAGAAA

MYC _FW: AGCTGCTTAGACGCTGGATT

MYC_REV: AAGTTCTCCTCCTCGTCGC

MYC-m6Apeak_FW: GCATACATCCTGTCCGTCCA

MYC-m6Apeak_REV: GTCGTTTCCGCAACAAGTCC

PES1_ex6_FW: ATGGGAAGAGCGAGTGGAAC

PES1_ex7_REV: GGGAAGGTGGAAAACAGGAAG

HPRT_FW: GCCATCACATTGTAGCCCTCTG

HPRT_REV: TTTATGTCCCCTGTTGACTGGTC

**Antibodies used in this study**

Anti-METTL3 [EPR18810] (Abcam), Anti-METTL14 antibody AMAb91276 (Atlas Antibodies), Anti-c-MYC (9E10) sc-40 (Santa Cruz Biotechnology, Dallas, Texas, USA), anti-PES1 A300-903A-T (Bethyl Laboratories), ﻿anti-WTAP 60188-1-Ig (Proteintech, Manchester, UK), anti-GAPDH BSM-33033M-HRP (Bioss, USA), Anti-Puromycin MABE343 - Clone 12D10 (Merck KGaA, Darmstadt, Germany).

**Real-time-PCR analysis**

RNA was extracted using the Quick RNA mini-prep kit (Zymo research, Irvine, CA USA). Reverse transcription was performed with the SuperScript VILO cDNA Synthesis Kit (Thermo Fisher Scientific, Waltham, MA USA). Real-time PCR was performed in triplicate using the SYBR™ Green PCR Master Mix (Thermo Fisher Scientific, Waltham, MA USA) on an Applied Biosystems 7500-Fast Real-Time-PCR System (Thermo Fisher Scientific, Waltham, MA USA). Relative expression levels of targets were determined using the comparative 2^ΔΔCt^ method. ActB mRNA was utilized as a reference.

**Immunofluorescence**

**﻿**10^6 cells were treated with Cytofix/Cytoperm Fixation/Permeabization Kit (#BD554714, Becton Dickinson). Cells were fixed in Fix Perm for 20 minutes at 4°C, rinsed with Wash Perm and incubated with the primary antibody anti-METTL3 (#15073-1-AP, Proteintech) and anti-GAPDH (#TA802519, OriGene). After two Wash Perm washes, cells were incubated with the secondary antibodies Alexa Fluor 555-labeled goat anti-mouse (#A-21422, Invitrogen) and Alexa Fluor 488-labeled goat anti-rabbit (#A-11034, Invitrogen). Nuclei were stained with Hoechst-33342 (Life Technologies). 20^4 cells were mounted in VECTASHIELD (#H-1000, Vector Laboratories). Images were acquired using a Zeiss LSM-900 confocal laser scanning microscope.
