# Supplementary material for: New insight into the catalytic -dependent and -independent roles of METTL3 in sustaining aberrant translation in chronic myeloid leukemia
Source: Cell Death Dis. 2021 Sep 24;12(10):870. doi: 10.1038/s41419-021-04169-7 (PMC8463696; doi:10.1038/s41419-021-04169-7)
Supplement: Supplementary file 2 — Supplemental Figure S1 [file 41419_2021_4169_MOESM2_ESM.pdf]

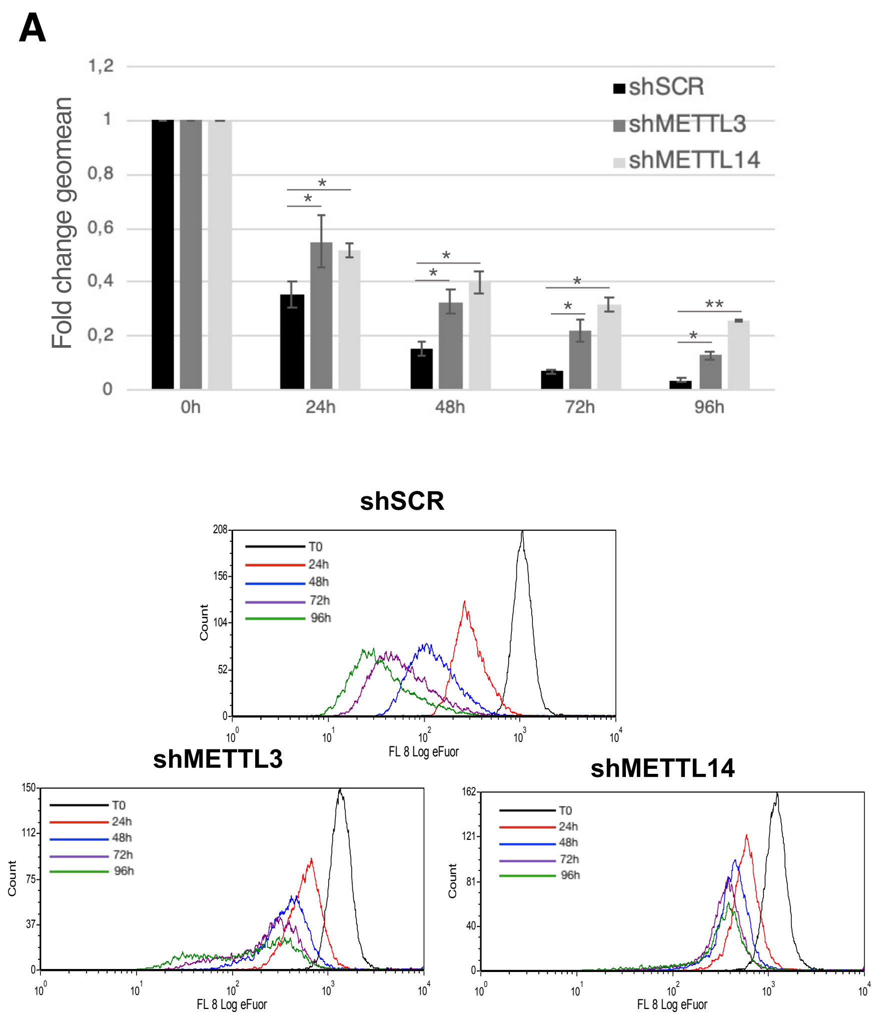

Supplemental Figure S1. Cell proliferation Efluor assay was performed using Efluor 670 Dye (Thermos Fisher Waltham, MA USA), a red fluorescent dye that can be used to monitor individual cell division. K562 cells were resuspended in PBS and Dye Efluor 670 was added at the 5μM final concentration. Cells were incubated 10 minutes at 37°C in the dark and washed with 0.5 ml of complete medium for 3 times. 10^5 cells were seeded in 12-well dish and after 72 h analyzed using an Epics XL Cytometer (Beckman Coulter). **a** transduced cells were stained after puromycin selection with the tracking dye and analyzed at the indicated time. The histogram represents the fold change of the geomean of fluorescence intensity. **b** representative FACS analysis. The ratio of each sample versus its experimental control was tested by two-tailed Student's t-test. * $p < 0.05$, ** $p < 0.01$.
