# Supplementary figures and images for: New insight into the catalytic -dependent and -independent roles of METTL3 in sustaining aberrant translation in chronic myeloid leukemia
Source: Cell Death Dis. 2021 Sep 24;12(10):870. doi: 10.1038/s41419-021-04169-7 (PMC8463696; doi:10.1038/s41419-021-04169-7)

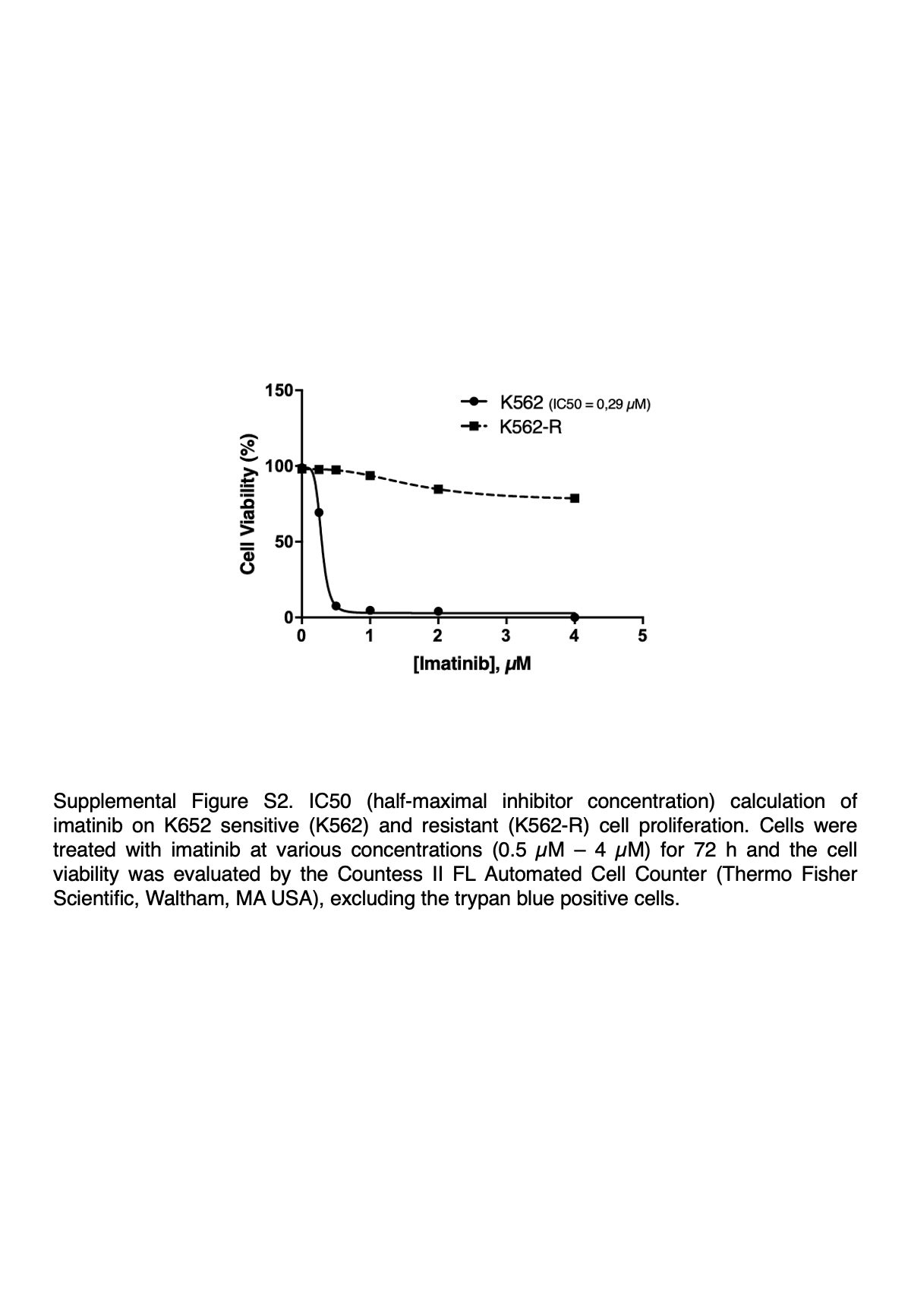

Supplement: Supplementary file 3 — Supplemental Figure 2 [file 41419_2021_4169_MOESM3_ESM.tif]

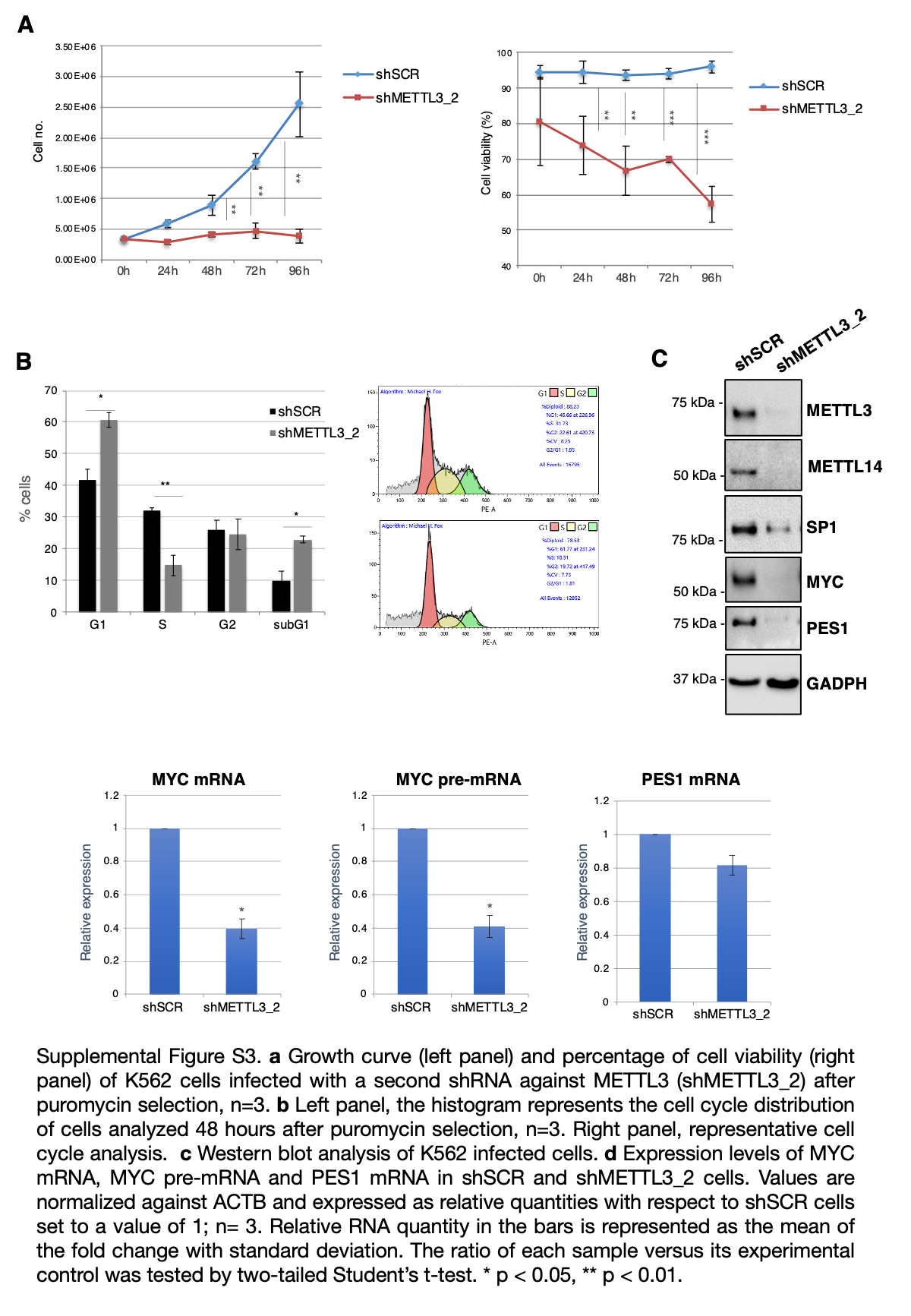

Supplement: Supplementary file 4 — Supplemental Figure 3 [file 41419_2021_4169_MOESM4_ESM.tif]

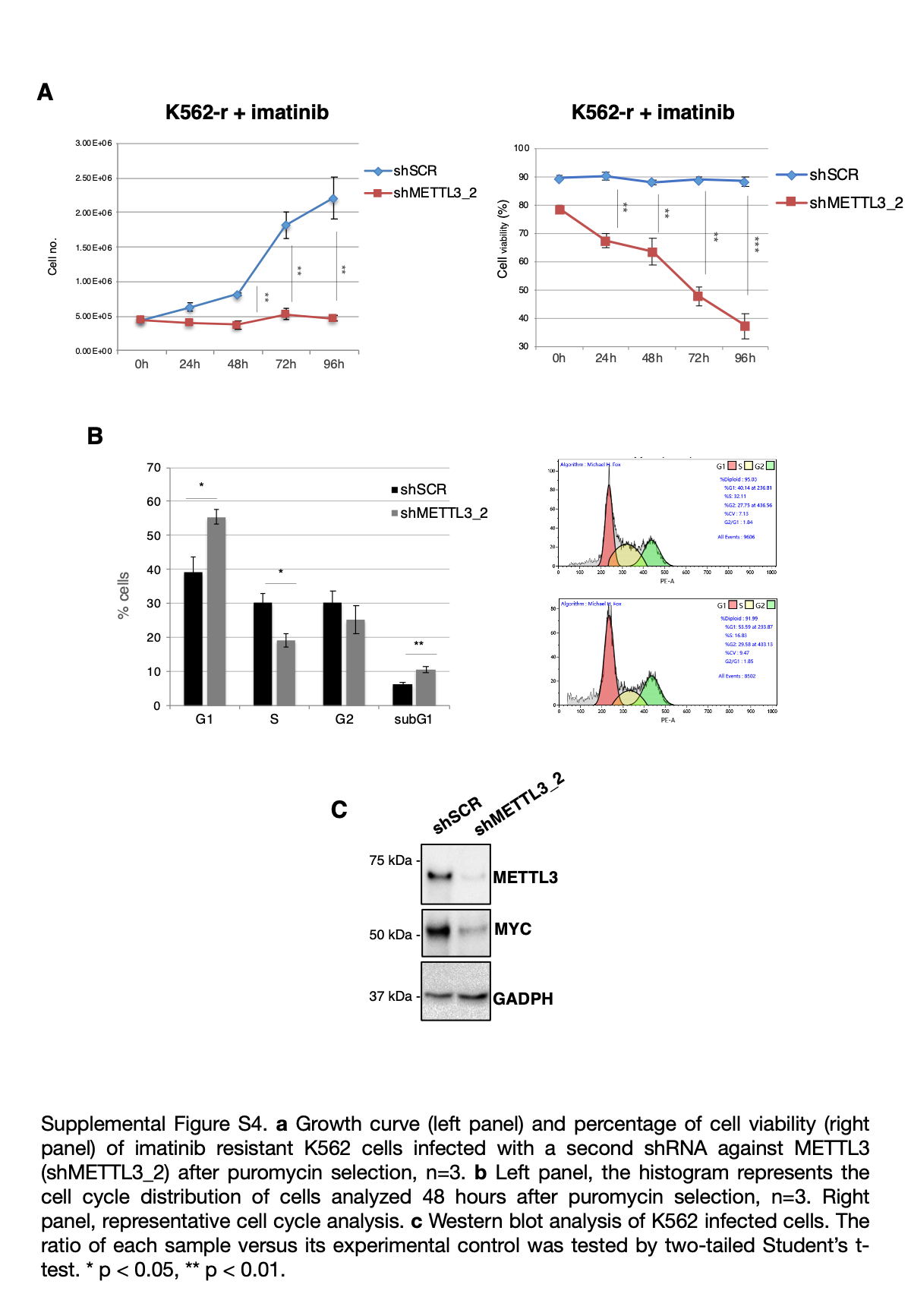

Supplement: Supplementary file 5 — Supplemental Figure 4 [file 41419_2021_4169_MOESM5_ESM.tif]

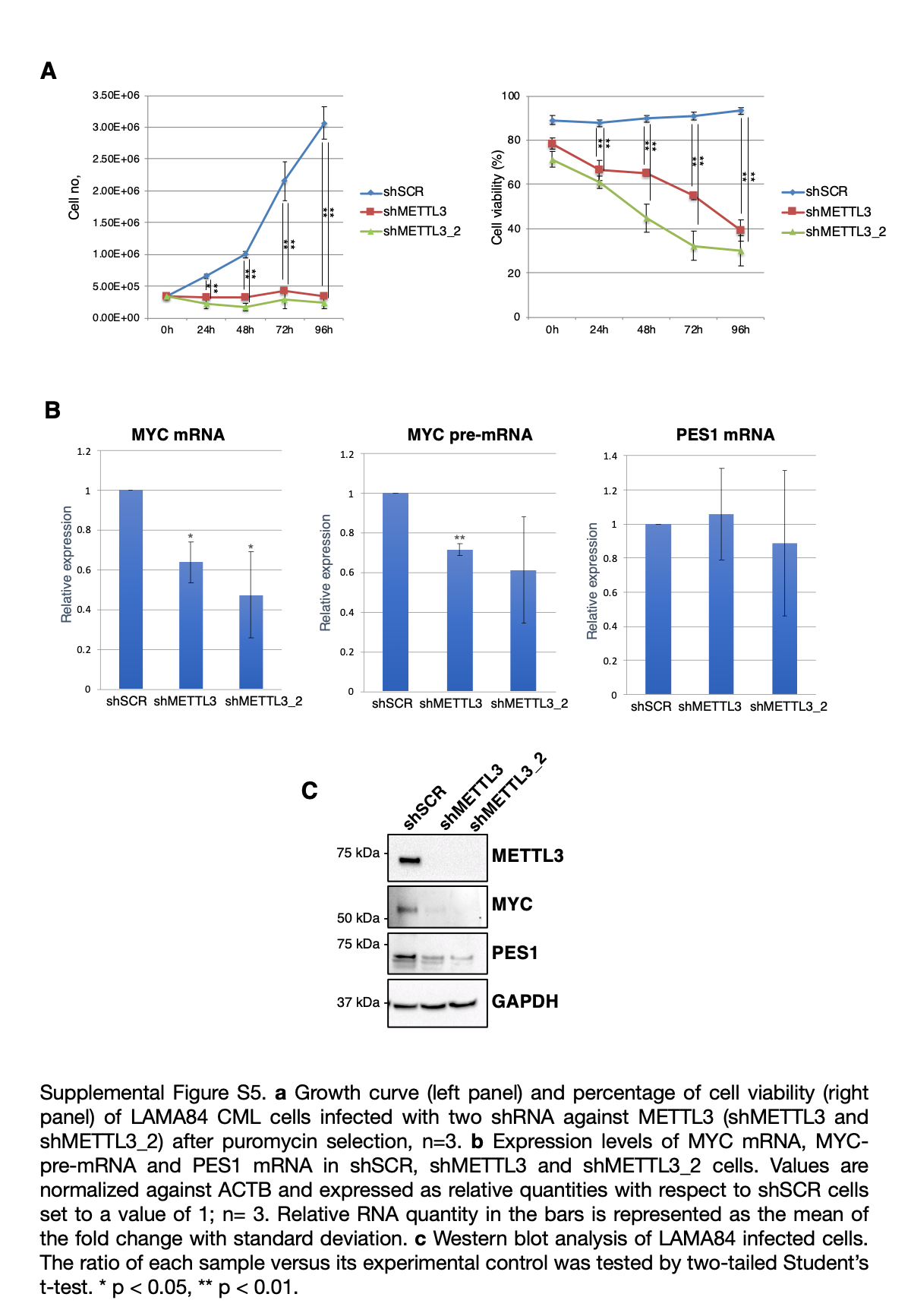

Supplement: Supplementary file 6 — Supplemental Figure 5 [file 41419_2021_4169_MOESM6_ESM.tif]

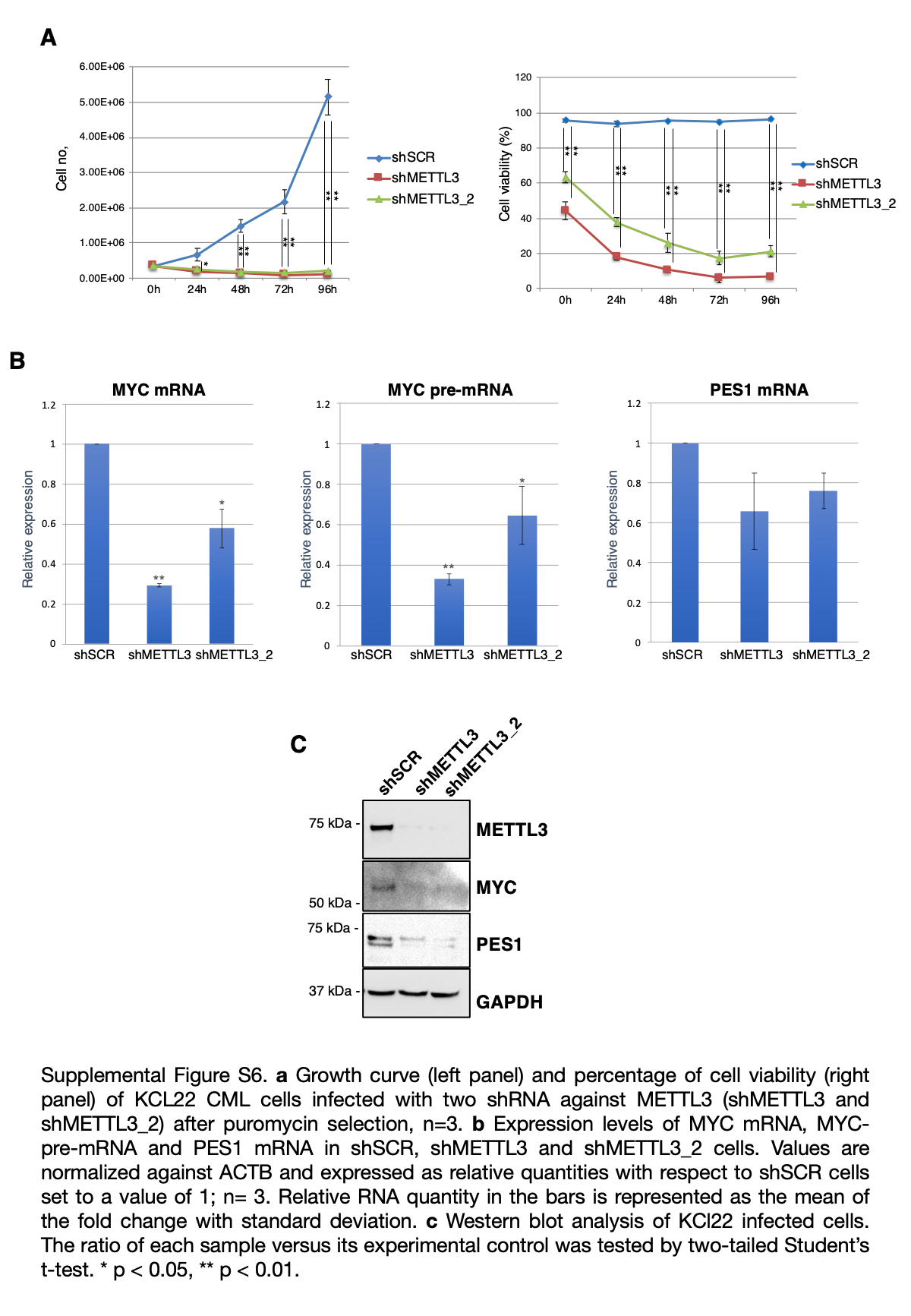

Supplement: Supplementary file 7 — Supplemental Figure 6 [file 41419_2021_4169_MOESM7_ESM.tif]

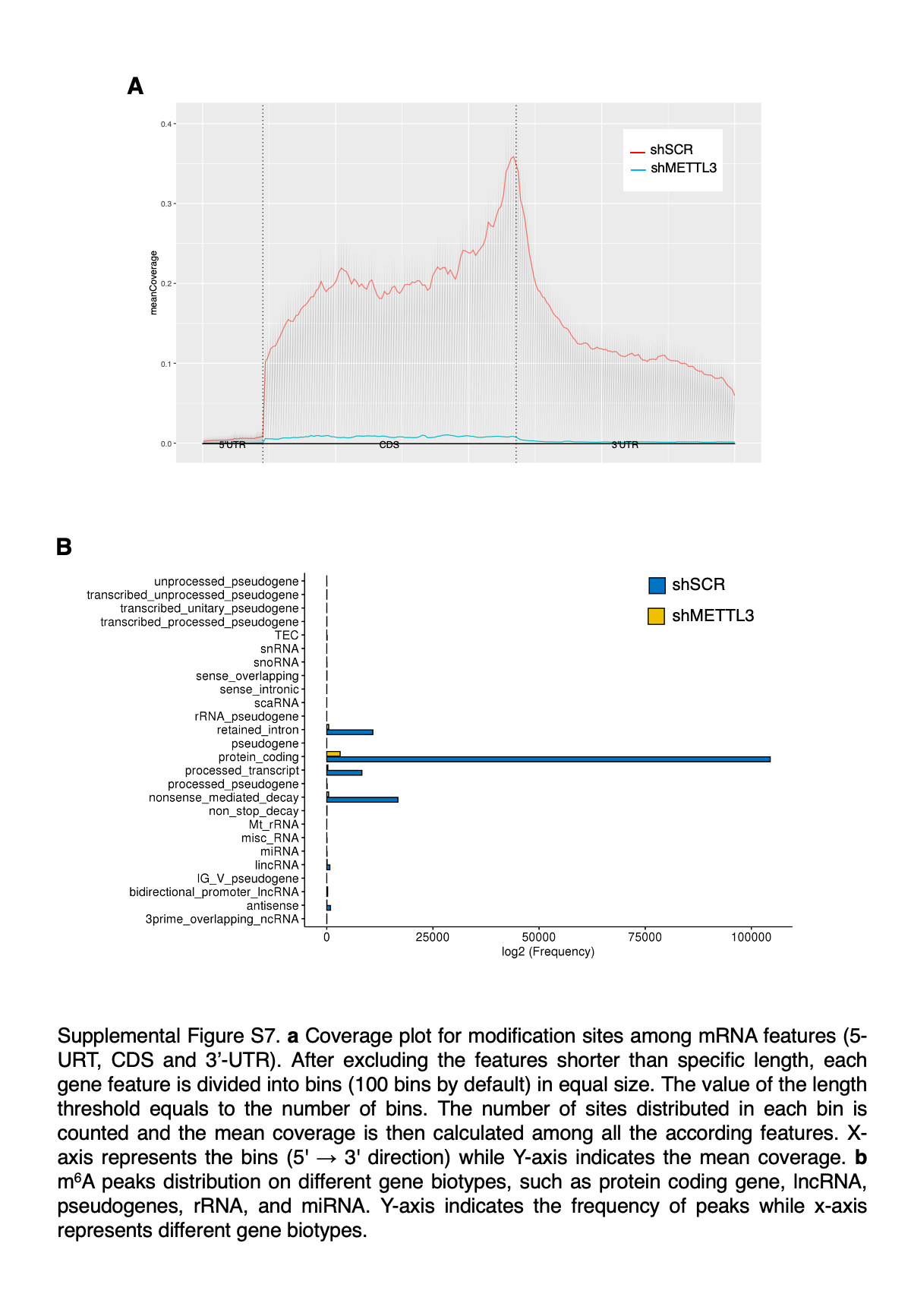

Supplement: Supplementary file 8 — Supplemental Figure 7 [file 41419_2021_4169_MOESM8_ESM.tif]

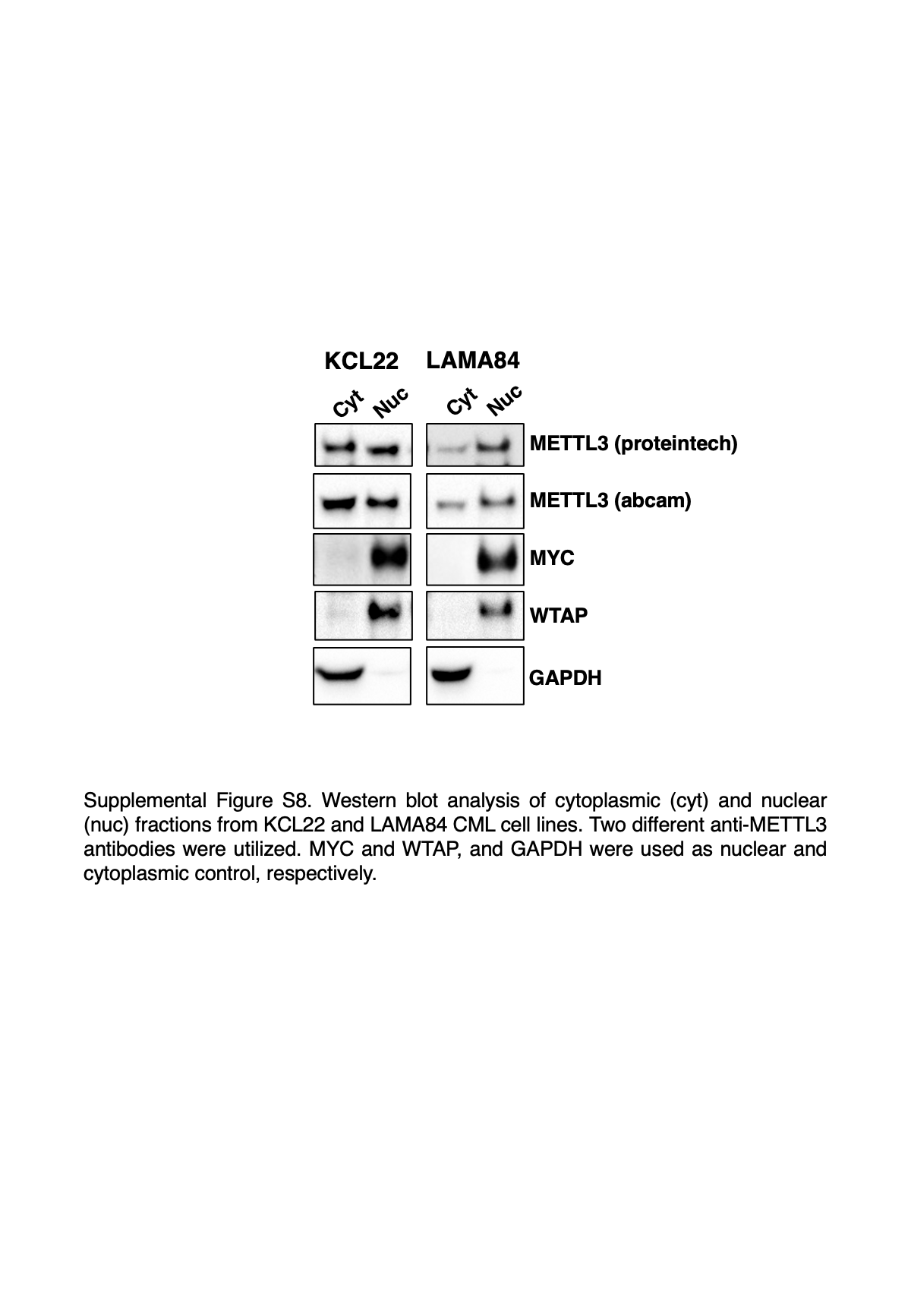

Supplement: Supplementary file 9 — Supplemental Figure 8 [file 41419_2021_4169_MOESM9_ESM.tif]

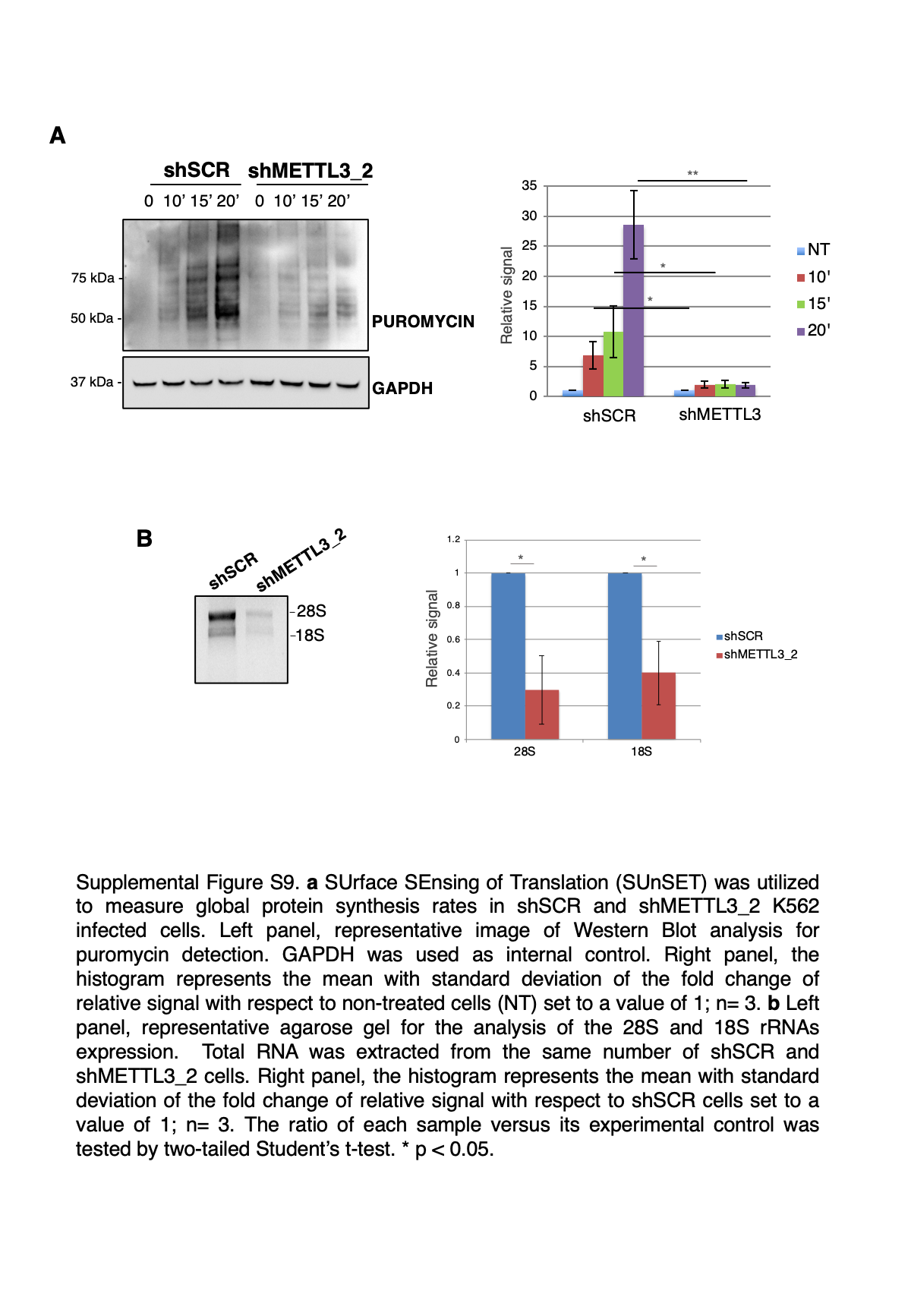

Supplement: Supplementary file 10 — Supplemental Figure 9 [file 41419_2021_4169_MOESM10_ESM.tif]

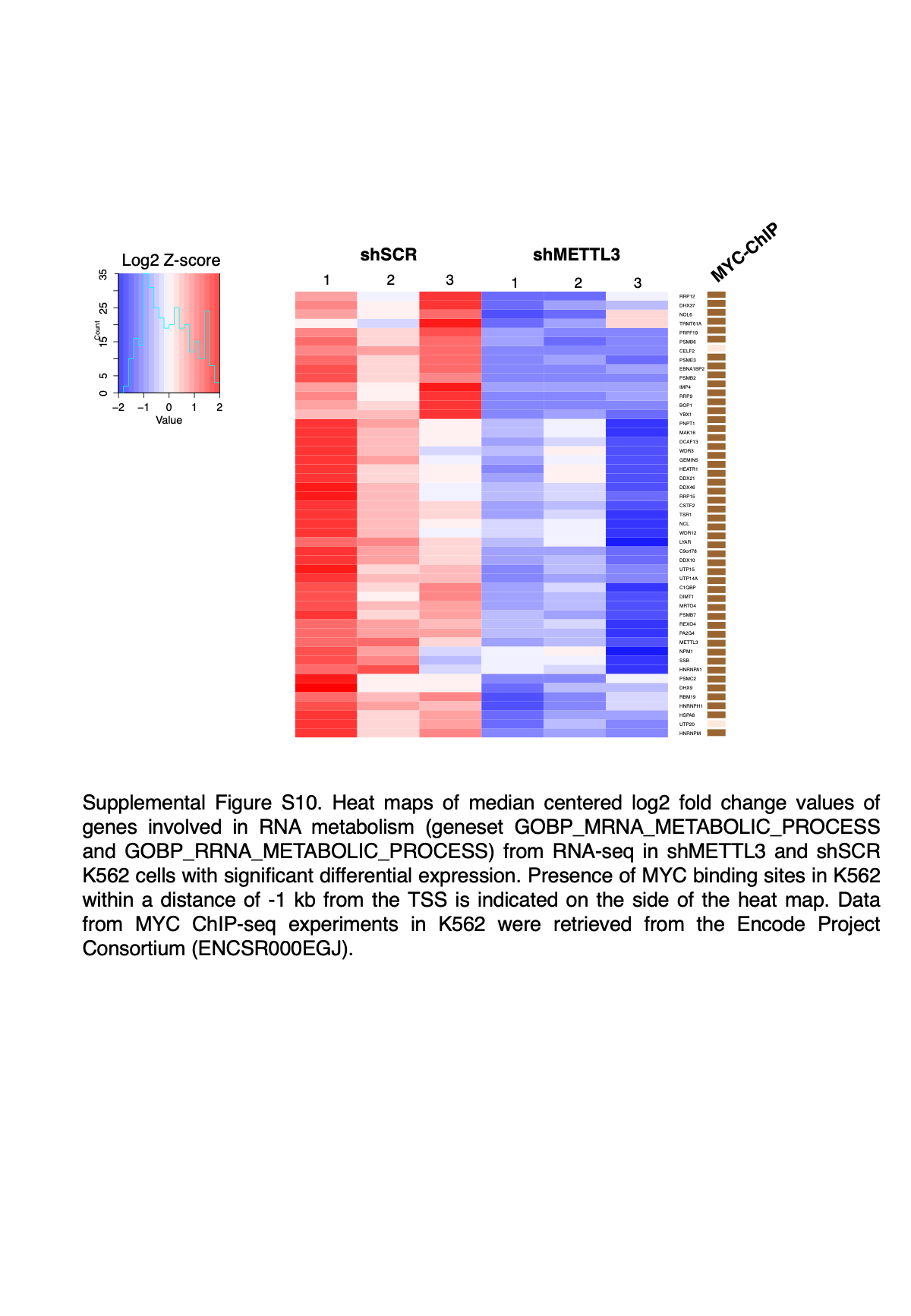

Supplement: Supplementary file 11 — Supplemental Figure 10 [file 41419_2021_4169_MOESM11_ESM.tif]

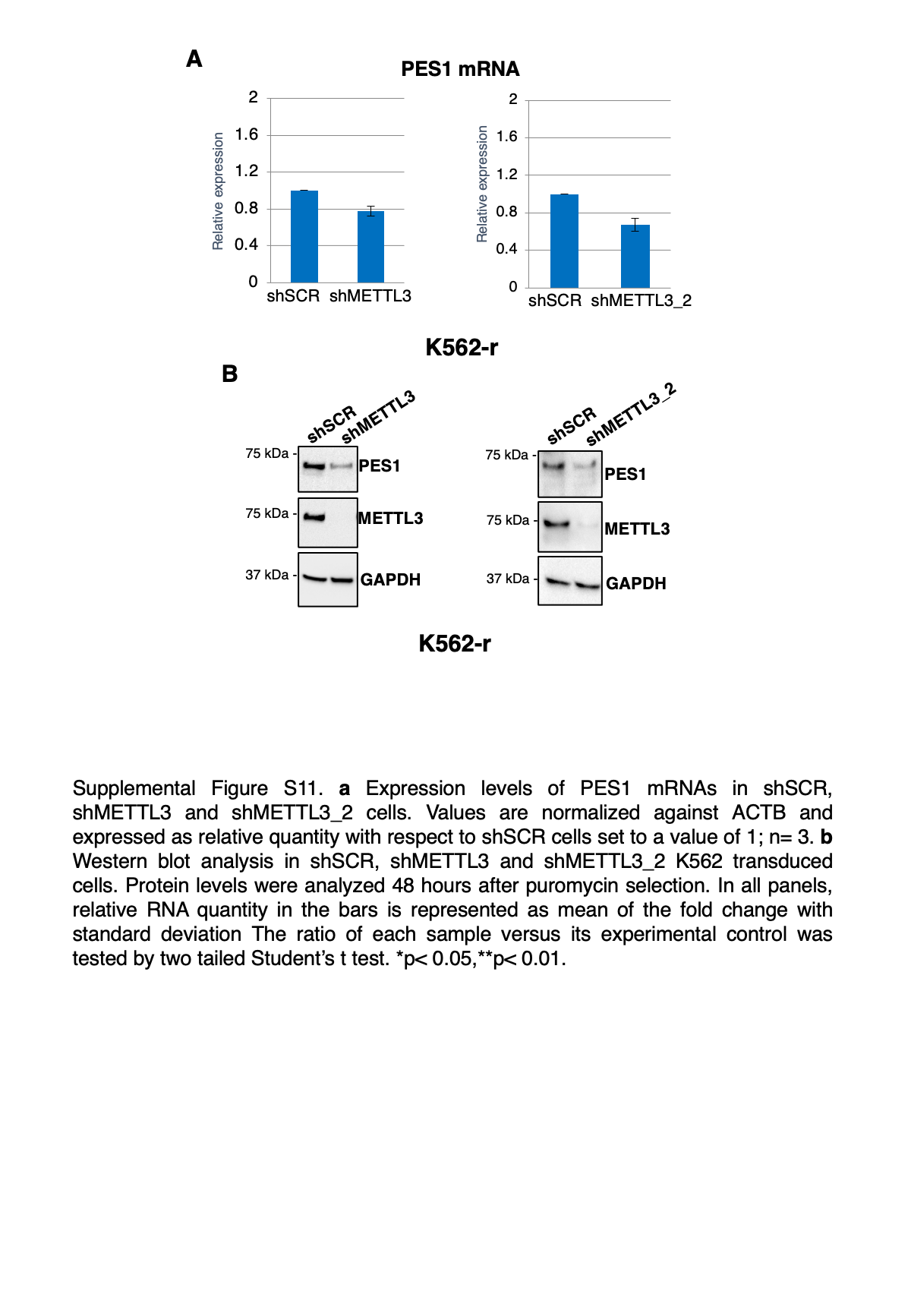

Supplement: Supplementary file 12 — Supplemental Figure 11 [file 41419_2021_4169_MOESM12_ESM.tif]
